# Human Acellular Amniotic Membrane as Skin Substitute and Biological Scaffold: A Review of Its Preparation, Preclinical Research, and Clinical Application

**DOI:** 10.3390/pharmaceutics15092249

**Published:** 2023-08-30

**Authors:** Yanqi Li, Siyu An, Chengliang Deng, Shune Xiao

**Affiliations:** 1 Department of Burns and Plastic Surgery, Affiliated Hospital of Zunyi Medical University, Zunyi 563000, China; yanqiliysj@sina.com; 2 The Collaborative Innovation Center of Tissue Damage Repair and Regeneration Medicine of Zunyi Medical University, Zunyi 563000, China; ansiyuzhengyi@sina.com

**Keywords:** human acellular amniotic membrane, skin substitute, skin dressing, tissue engineering, biological scaffold

## Abstract

Human acellular amniotic membrane (HAAM) has emerged as a promising tool in the field of regenerative medicine, particularly for wound healing and tissue regeneration. HAAM provides a natural biological scaffold with low immunogenicity and good anti-infective and anti-scarring results. Despite its potential, the clinic application of HAAM faces challenges, particularly with respect to the preparation methods and its low mechanical strength. This review provides a comprehensive overview of HAAM, covering its preparation, sterilization, preclinical research, and clinical applications. This review also discusses promising decellularization and sterilization methods, such as Supercritical Carbon Dioxide (SC-CO_2_), and the need for further research into the regenerative mechanisms of HAAM. In addition, we discuss the potential of HAAM as a skin dressing and cell delivery system in preclinical research and clinical applications. Both the safety and effectiveness of HAAM have been validated by extensive research, which provides a robust foundation for its clinical application.

## 1. Introduction

Traumatic injuries and their consequential scars and complications continue to be significant clinical challenges affecting millions of people worldwide annually. For example, the World Health Organization (WHO) reports that about 11 million people annually sustain burn injuries, leading to approximately 180,000 fatalities [1]. Non-fatal burns are a leading cause of morbidity, often leading to prolonged hospitalization, disfigurement, and disability, as well as resulting stigmas and rejection. For deep or even full-thickness skin wounds, split thickness skin grafting (STSG) is the gold standard [2], but it causes new damage to the donor site and is limited by the skin source and availability of the donor site. Furthermore, the primary culture steps necessary for this treatment can cause delays to its availability [3]. This has led to the exploration and utilization of alternative solutions. Currently, different skin substitutes are being used in clinics, mainly in regard to allogeneic skin grafts, xenografts, and synthetic skin substitutes [4]. However, these substitutes come with their unique challenges. Allogeneic skin’s primary drawback is the potential immune response, which may cause tissue rejection. Its supply, constrained by human donors, could be limited, and it carries a disease transmission risk. Similarly, xenografts, though temporarily usable due to their lower immunocompatibility, have limited availability and pose a disease transmission risk [5]. Synthetic skins, while avoiding biological risks, may not perfectly replicate human dermis’ complexity and function [6]. Their biodegradability and mechanical attributes might also not align with natural skin characteristics. Therefore, there is a critical need for innovative wound healing therapies.

With the limitations of these existing therapies in mind, the attention of many researchers has been drawn towards the potential of biological dressings, which are materials derived from living sources that are used to cover wounds. Amnion membrane (AM) is one of the oldest skin substitutes. With a thickness of 20–50 µm and a five-layered structure, AM has been utilized as a biological dressing for burn wounds since 1910 [7]. AM has characteristics which promote epithelization, angiogenesis, and the inhibition of scar formation [8]. In addition, AM tissue also secretes leukocyte protease inhibitor and elastin, which have antibacterial and anti-inflammatory effects [9]. Therefore, AM has been explored for various clinical cases, including lower extremity venous ulcers [10], ocular injury trauma [11], dental defect trauma [12], and vaginal reconstruction surgery [13]. Despite its promising characteristics, its use is hampered by several challenges, including bacterial and viral contamination, the potential risk of disease transmission (e.g., HIV, Hepatitis B and C), and difficulties in storage. In response to these challenges, a novel solution has emerged in the form of human acellular amniotic membrane (HAAM), which offers several key advantages.

HAAM is an ideal skin substitute and natural biological scaffold with the following characteristics: (1) abundant sources, convenient preparation, cost effectiveness, and wide availability; (2) excellent biocompatibility and biodegradability [14]; (3) the fact that it retains the extracellular matrix (ECM) structure for cell adhesion and growth; (4) the fact that it contains biological factors for promoting cell adhesion, growth, proliferation, and differentiation [15]; (5) the fact that it has antibacterial and anti-inflammatory activity [16]; (6) the fact that it offers good flexibility and can be used for covering irregular wound surfaces; and (7) the fact that it promotes the regeneration of skin appendages [17]. As a surgical patch and skin substitute with good biocompatibility, natural HAAM has wide applications, including body wall repair [18], cardiac patches [19], skin wound dressings [20], etc. Furthermore, HAAM can also be used as a tissue scaffold and acts as a cell delivery vehicle, which can play a role in the delivery of drugs, autologous stem cells, or growth factors, providing a controlled release of these substances to promote healing and regeneration [21].

HAAM does not contain amniotic epithelial cells but preserves the amniotic basement membrane and dense layer, reducing the risk of immune rejection while preserving the beneficial properties of the amniotic membrane. Its main components are collagen types III, IV, and V; proteoglycans; and glycoproteins, which form a specialized ECM [22]. Through decellularization, cellular components that cause immunologic rejection are removed, preserving the morphology, three-dimensional (3D) structure, and composition of the ECM [23]. The resulting HAAM retains the key properties of AM, including low immunogenicity, and provides anti-infective and anti-scarring effects, which are highly desirable for skin substitutes [24]. Moreover, HAAM presents a more practical storage solution, bypassing the need for −80 °C temperatures and sterile vials, consequently reducing medical costs. Furthermore, HAAM serves as a novel cell transplantation scaffold, simulating the cellular microenvironment and promoting epithelial cell migration and proliferation, thereby potentially accelerating the healing of chronic wounds [25]. As a biomaterial, HAAM thus offers unique advantages in both wound care and regenerative medicine, meriting further investigation.

**Figure 1:**
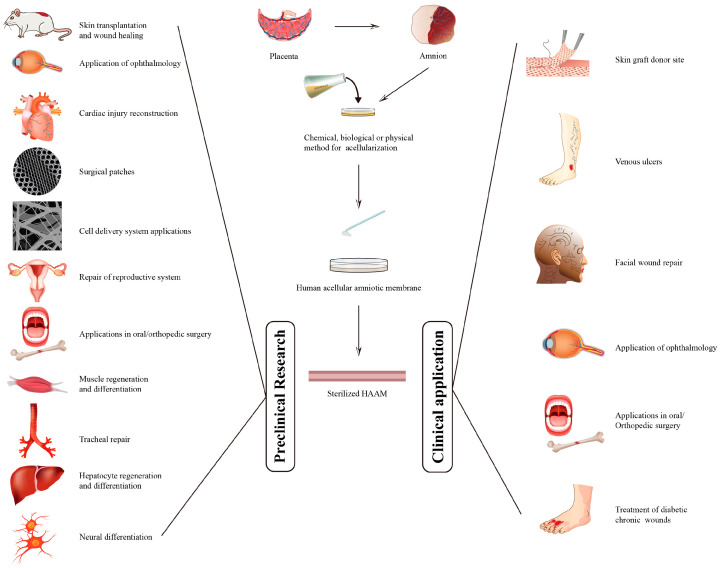
Preparation process, preclinical research, and clinical applications of human acellular amniotic membrane.

Despite the promising results of HAAM in wound healing and regenerative medicine, our understanding of its mechanism of action, potential applications, and potential challenges is far from complete. Various individual studies have explored aspects of HAAM, but there has yet to be a comprehensive review that integrates these diverse findings and provides a holistic overview. This review aims to fill this gap by providing a comprehensive overview of the developments in the field of HAAM as a skin substitute and cell scaffold, including its preparation, the mechanisms underlying its wound healing properties, its preclinical applications, and the outcomes of clinical research. By doing so, we aim to underscore the benefits and potential of HAAM in enhancing wound care and improving patient outcomes, thereby advancing our understanding of HAAM and inspiring further research and innovation in this area. Figure 1 presents the preparation process, preclinical research, and clinical applications of human acellular amniotic membrane.

## 2. Materials and Methods

This review was conducted through a comprehensive search of several academic databases, including Google Scholar, PubMed, Science Direct, and Web of Science. The search was focused on peer-reviewed research articles published between 2001 and 2023.

The search strategy involved the use of several key terms related to the use of HAAM as a skin substitute and biological scaffold. The keywords used in the search included ‘human acellular amniotic membrane’, ‘acellular amniotic membrane’, ‘decellularized human amniotic’, ‘amniotic membrane wound healing’, and ‘preparation and sterilization of amniotic membrane’. Both preclinical and clinical studies were considered for inclusion in the review. Studies were excluded if they did not focus on HAAM or if they were not published in English.

The selected articles were then thoroughly reviewed using a standardized data extraction form which, in the context of this study, refers to a set of specific tables created to uniformly capture relevant information. These tables were designed with particular fields to systematically extract the critical details of each study, including details on the preparation of HAAM, preclinical research involving HAAM, and the clinical applications of HAAM. The extracted information was then synthesized and analyzed to provide a comprehensive overview of the current state of research on the use of HAAM as a skin substitute and biological scaffold.

## 3. Preparation and Sterilization of HAAM

The preparation and sterilization of HAAM are critical steps in its use as a tissue engineering scaffold. The methods used can significantly affect the complex structure, mechanical strength, and biochemical performance of HAAM. Therefore, it is essential to understand the different methods available and their respective advantages and disadvantages. As shown in Table 1, we have summarized the representative methods for the preparation and sterilization of HAAM, along with the advantages and disadvantages of each method.

### 3.1. Preparation of HAAM

This subsection will discuss the different methods used to prepare HAAM, including chemical, biological, and physical methods. Each method will be evaluated based on its efficiency, cost, safety, and impact on the properties of HAAM.

#### 3.1.1. Chemical Methods

Chemical methods are commonly used in the preparation of HAAM due to their efficiency in solubilizing cellular membranes and nuclear material, aiding in the removal of cellular components from the AM. These methods involve the use of various chemical agents, each with their unique advantages and disadvantages.

Surfactants [14], which are compounds that lower the surface tension between two liquids or a liquid and a solid, are frequently used in chemical preparation methods. Among the surfactants, sodium dodecyl sulfate (SDS) is the most frequently used due to its effectiveness in solubilizing cellular components and high biodegradability of >90%. However, its use requires careful handling due to its potential to cause excessive tissue damage at high concentrations. For instance, a study by Xing et al. [26] found that high SDS concentration significantly reduced the ECM content and impacted their mechanical strength. The elastic and viscous moduli of the ECM decreased by approximately 80% and 62%, respectively. To mitigate this, SDS is often used in combination with Triton X-100, which is less damaging to the ECM [27].

Acid and base solutions, such as peracetic acid, are also used in the chemical preparation of HAAM [28]. These solutions alter the pH environment of the tissue, leading to the denaturation and solubilization of cellular proteins. This process aids in the removal of cellular components from the AM, making these solutions a useful tool in the preparation of HAAM. For example, in the quest for optimal decellularization methods for Human Amniotic Membrane (HAM), a study conducted by Milan et al. in 2020 found that HAAM prepared with 2 M peracetic acid showed superior results in GAG quantification, DNA isolation and quantification, histological assessment, collagen analysis, a cell–tissue interaction study, and with respect to cytotoxicity. The use of peracetic acid in this study effectively preserved the ECM’s natural components and structure while efficiently removing cell fragments [28]. However, care must be taken to neutralize the pH after treatment to prevent damage to the extracellular matrix. In summary, while acid and base solutions are effective in preparing HAAM, their use requires careful handling to ensure the preservation of the HAAM’s structural integrity and biological functionality.

Both hypotonic and hypertonic saline solutions play roles in the chemical preparation of HAAM. Hypotonic saline, with its lower salt concentration compared to body cells, can cause water to rush into the cells due to an osmotic imbalance. This leads to cell swelling and can eventually result in cell lysis or rupture, aiding in the decellularization process [29]. Hypertonic saline, a solution with a higher concentration of salt than normal body cells, is another agent used in the chemical decellularization of HAAM [27]. The high salt concentration in the solution creates an osmotic pressure difference across the cell membranes, causing water to move out of the cells, leading to cell shrinkage and, ultimately, cell lysis or rupture. The exact concentration of the hypertonic saline and the duration the membranes are immersed can vary, but often, a solution with a concentration between 1.0 M and 1.5 M is used, and the duration could range from a few hours to a few days. This method is advantageous as it does not leave any residual chemicals that could potentially cause adverse reactions.

Lastly, chelating agents such as ethylenediaminetetraacetic acid (EDTA) are used in the chemical preparation of HAAM [30]. These agents work by binding to divalent cations, which are necessary for the structural integrity of cell membranes. This leads to cell membrane destabilization and the removal of cellular components, so the method cannot be used for decellularization alone [31].

While chemical methods are efficient in preparing HAAM, it is crucial to thoroughly rinse the membrane after decellularization to remove any residual chemicals. These chemicals, if not properly removed, could potentially cause adverse reactions upon transplantation. Furthermore, each chemical agent has its advantages and disadvantages, and the choice of agent depends on the specific requirements of the application. In summary, while chemical decellularization methods have been widely used, they have certain limitations, including long processing times, potential for residual chemicals, cytotoxicity, and potential damage to the ECM. Therefore, the choice of chemical method should be carefully considered based on the specific requirements of the application and the need to balance efficiency with the preservation of the HAAM’s structural integrity and biological functionality.

#### 3.1.2. Biological Methods

Biological methods for the preparation of HAAM leverage the action of enzymes to strip the tissue of its cellular components. This approach is considered due to its potential for better biocompatibility compared to chemical and physical methods. Some common enzymes used in this process include trypsin [32], DNAase [33], and lipase [27], with each possessing distinct characteristics and effects on the tissue.

Trypsin, a proteolytic enzyme, is the most commonly employed biological agent for HAAM preparation due to its potent and reliable digesting ability [32]. Trypsin acts by breaking down proteins, which aids in the disruption and removal of cells from the tissue. Its high biocompatibility makes it a favorable choice for biological decellularization. However, there are some inherent drawbacks to using trypsin. Overextended exposure to trypsin can lead to the degradation of key elements in the HAAM, including growth factors, collagen, and other integral components of the ECM [34]. This is a critical concern as these components are vital for HAAM’s regenerative properties. Lipase, on the other hand, specifically targets and breaks down lipids. However, studies have shown that lipase is less effective in preparing HAAM as it does not manage to fully digest all lipids present in the tissue, leaving remnants that could potentially induce an immune response or alter the tissue’s properties [27]. DNAase is another enzyme used for decellularization that works by breaking down DNA into smaller fragments, which can then be more easily washed away. However, similar to lipase, its efficacy is questionable, as it may not fully degrade and remove all DNA present in the tissue [33].

In summary, while biological or enzymatic decellularization methods may not achieve complete cell removal and can potentially damage the basement membrane and ECM, they continue to be explored due to their potential for better biocompatibility compared to chemical and physical methods. The ideal decellularization method must achieve a fine balance. It should efficiently remove all cellular components to minimize the risk of adverse immune responses, but at the same time, it should preserve the ECM to the maximum extent possible, as its preservation is key to the successful use of HAAM in regenerative medicine.

#### 3.1.3. Physical Methods

Physical methods for HAAM decellularization, including the freeze–thaw method and mechanical scraping, primarily function by applying physical stress to disrupt cells and facilitate their removal. These methods are considered due to their potential to minimize chemical residues and maintain the structural integrity of the HAAM.

The freeze–thaw method utilizes temperature changes to lyse or rupture cells. This method involves repeatedly freezing and then thawing the amniotic membrane [35]. During freezing, ice crystals form within and around cells, which disrupts their structure. Subsequent thawing then causes the cells to burst due to osmotic pressure changes. This process inflicts minimal damage to the AM matrix, and the 3D scaffold structure of the ECM is largely preserved [36]. Nevertheless, the freeze–thaw method does come with certain limitations. Rapid temperature changes are required to effectively lyse the cells, and this can be technically challenging to achieve. Moreover, the ice crystals that form during the process may cause varying degrees of damage to the ECM [37].

Mechanical scraping is another physical method used in the decellularization process [25]. This method involves physically scraping the surface of the amniotic membrane to remove cells and cell debris. Although mechanical scraping can be effective in aiding cell removal, the intensity of the scraping can potentially harm the integrity of the basement membrane and ECM, both crucial for the biological function of the HAAM [38].

A novel technique for creating tissue scaffolds by decellularizing live tissues is nonthermal irreversible electroporation (NTIRE). This approach damages cells at the nanoscale, preserving the ECM, and subsequently leverages the body’s immune response for decellularization. The resulting scaffold retains its native ECM structure, supports recellularization, and ensures functionality. While NTIRE offers precise tissue decellularization, challenges arise from its reliance on host response, potential for unintended tissue targeting, equipment complexity, unknown long-term effects, and scalability limitations [39].

High Hydrostatic Pressure (HHP) is also a physical technique that involves applying intense pressure to decellularized tissues. Its merits include non-thermal processing, the preservation of structural integrity, and broad-spectrum microbial inactivation without chemical residues. Conversely, its challenges include high equipment costs, potential structural alterations at extreme pressures, efficacy variability based on product characteristics, and significant energy consumption [40].

Given the limitations of both physical methods, they are often not used as standalone decellularization techniques; instead, they are commonly employed as auxiliary decellularization methods in combination with other strategies (e.g., enzymatic or chemical decellularization). This combination approach helps ensure the more complete removal of cellular components, minimizing immunogenicity while retaining the desirable properties of the ECM for therapeutic applications. Despite the challenges, physical methods remain an important part of the toolkit for HAAM decellularization, contributing to the optimization of the overall process.

### 3.2. The Sterilization of HAAM

The sterilization of HAAM is a crucial step in its preparation. This process is necessary to eliminate any residual bacteria, viruses, and chemical/biological reagents that could cause cytotoxic effects or transmit infections. Several sterilization methods are commonly used, each with their own advantages and disadvantages.

(1) Irradiation, specifically γ-ray irradiation, is widely used in clinical practice [41]. Dry irradiated AM can accelerate wound epithelialization, promote healing, and reduce the frequency of dressing changes as well as pain, scar formation, and hospitalization time [41]. However, Singh et al. found that high doses of γ-rays (25 kGy), while completely sterilizing the membrane, caused the destruction and degradation of the AM basement layer [42]. Höynck et al. showed that strong radiation causes the destruction of the AM epithelium, degeneration of vacuolation, and dissolution of the connective tissue layer into single fibrous bundles [43].

(2) Ethylene oxide is another sterilization method that can eliminate all microorganisms and penetrate objects with an irregular morphology, complex structure, and impermeability. Despite its disadvantages of flammability, explosiveness, and toxicity, Xiao et al. reported that HAAM was sterilized with ethylene oxide without damaging the porous morphology of the HAAM scaffold [44].

(3) Peracetic acid is a widespread antimicrobial agent with strong antibacterial, antifungal, antiviral, and anti-spore effects. It not only effectively removes DNA during acellular treatment but also has no effect on the composition of AM when used for sterilization and disinfection. However, the use of peracetic acid-sterilized AM can result in changes in its structure, such as epithelial surface folding and flattening and microvilli condensation [43].

(4) SC-CO_2_, a supercritical fluid, has been widely used as an extractant [45]. It is an emerging technology that is currently used in the sterilization of medical instruments, implants, and human-transplanted tissues [43]. SC-CO_2_-sterilized AM overcomes the limitations of other sterilization methods, such as damage to the structure of the ECM, cytotoxicity, and insufficient sterilization. Wehmeyer et al. demonstrated that the tissue structure and collagen composition of AM were unaffected by sterilization with SC-CO_2_, while its biological properties and biocompatibility were maintained [46].

**Table 1:** Preparation and sterilization of HAAM.

Method	Advantage	Disadvantage	Reference
**Chemical method**			
surfactant	completely decellularized and degraded DNA	low efficiency; damages ECM and easily leaves chemical remains	[14]
acid/base solution	complete decellularization and high efficiency	damages ECM and growth factors	[28]
Hypotonic/hypertonic saline	gentle	low efficiency; not completely decellularized	[27,29]
chelating agent	gentle	not completely decellularized; combination application needed	[30]
**Biological method**			
trypsin	completely decellularized, high efficiency and high biocompatibility	degraded ECM; high costs and influenced by temperature, environment, and pH	[34]
DNAase	enzymatic digestion of cell nucleus	low efficiency; not completely decellularized	[33]
lipase	hydrolysis lipids	low efficiency; not completely decellularized	[27]
**Physical method**			
freeze–thaw	Less damage to ECM	Low efficiency; high requirement regarding temperature change rate	[36]
mechanical scraping	most commonly used auxiliary methods	not completely decellularized	[25]
nonthermal irreversible electroporation	preservation of ECM, no thermal damage, facilitating recellularization	reliance on host response; equipment complexity	[39]
High hydrostatic pressure	Non-Thermal Process, effective, No Chemical Residues	expensive, product variability, potential for uneven pressure distribution	[40]
**Sterilization of HAAM**			
irradiation	complete sterilization	damages ECM structure	[42]
Ethylene oxide	complete sterilization and elimination of all microorganisms	flammable, explosive, and toxic	[44]
Peracetic acid	complete sterilization	interfere tissue structures	[43]
Supercritical carbon dioxide	complete sterilization, high biocompatibility, and environmental friendliness	accurate control of pressure rate	[45]

## 4. Mechanisms Underlying the Regenerative Ability of HAAM

HAAM possesses unique biological properties, and its potential for wound healing and regenerative medicine has been widely recognized. Its applications have been documented in various fields, including skin transplantation and wound healing [15], ophthalmology [47], orthopedics [48], cell culture matrices [32], cell delivery systems [14], cardiac injuries [49], male and female reproductive systems [50], and liver [51]. However, the exact mechanisms that underpin HAAM’s regenerative capacity are not yet fully understood.

### 4.1. The Role of Cells and Growth Factors in HAAM-Mediated Healing

The regenerative abilities of HAAM are multifaceted and hinge on the mechanism of creating a supportive scaffold for reparative cells at the wound site. One of the key mechanisms behind HAAM’s regenerative ability is its role in promoting the migration of reparative cells to the wound site. Macrophages are a type of white blood cell critical to both inflammation and wound healing. Upon a wound’s occurrence, HAAM attracts these macrophages to the site, where they perform crucial roles in the healing process. Macrophages initiate phagocytosis, a process that involves engulfing and digesting cellular debris and pathogens, thereby preventing potential infections and clearing the wound site of any obstructive debris. In addition to this, macrophages secrete various cytokines and growth factors, including Transforming Growth Factor-beta (TGF-β). These molecular compounds guide the healing process by regulating the activity of the other cells involved in wound healing and tissue regeneration. For instance, TGF-β aids in promoting cell migration and proliferation at the wound site, stimulates the production of extracellular matrix proteins to provide structural support to the healing tissue, and assists in managing the immune response. Therefore, through its role as a scaffold for the migration and activity of macrophages and other reparative cells, HAAM plays a pivotal role in the process of wound healing and tissue regeneration [52,53,54].

Another key mechanism is the promotion of fibroblast migration to the wound site. Fibroblasts are cells that synthesize the extracellular matrix and collagen and are vital for tissue repair and regeneration [55]. The migration of fibroblasts to the wound site is encouraged by the physical properties and biochemical composition of HAAM. The HAAM provides an abundant source of collagen, a key component required by fibroblasts to synthesize new tissue structures. Its three-dimensional structure provides sufficient space for fibroblast growth, and the tensile strength of the HAAM, enhanced by its collagen-rich composition, provides a supportive environment for fibroblasts to function effectively. They essentially construct a new framework upon which new cells can grow and flourish, which is essential for wound healing. Once at the wound site, fibroblasts engage in the synthesis of the extracellular matrix, a complex mixture of proteins and carbohydrates that provide a structural scaffold to tissues. Furthermore, fibroblasts produce collagen [56], a protein that is fundamental for the formation of granulation tissue (new connective tissue) and tiny blood vessels that form on the surfaces of a wound during the healing process. Collagen also aids in wound contraction, a process that helps to reduce the size of a given wound.

HAAM also contains growth factors such as epidermal growth factor (EGF) [57], vascular endothelial growth factor (VEGF) [51], and fibroblast growth factor (FGF) [58]. These growth factors promote cellular proliferation and migration, angiogenesis (the development of new blood vessels), and the remodeling of the extracellular matrix, thereby expediting the healing process.

In essence, HAAM promotes wound healing through a variety of mechanisms, including the attraction of macrophages and fibroblasts to the wound site, the provision of a supportive environment for these cells, and the presence of growth factors that foster cellular proliferation and migration. Understanding these mechanisms can help researchers develop more effective treatments for a variety of conditions.

### 4.2. HAAM as a Bioactive Scaffold and Cell Delivery System

The use of the HAAM as a cell delivery system is a significant development in the field of tissue engineering and regenerative medicine [14,59]. This application allows for the direct delivery of therapeutic cells to the wound site, enhancing the body’s natural healing response and potentially improving overall outcomes in wound healing and tissue regeneration.

HAAM acts as a biological vehicle or “carrier”, delivering cells such as stem cells, fibroblasts, or other progenitor cells directly to the wound or damaged tissue. These cells can then integrate into the local tissue environment, proliferate, and differentiate into the required cell types to facilitate repair and regeneration. The use of HAAM as a cell delivery system leverages its high biocompatibility and the supportive environment it provides for cell growth and differentiation [60]. As a natural, biological material, HAAM offers an environment that is conducive to cell survival and function. It can support the attachment, growth, and differentiation of the delivered cells, thus enhancing the overall effectiveness of the cell therapy.

This strategy of combining HAAM with other materials or cells can be tailored to meet the specific needs of the wound or tissue defect. For example, HAAM can be combined with growth factors or other bioactive molecules to enhance the healing response. Alternatively, it can be combined with specific cell types that are chosen based on the specific tissue that is being repaired. Stem cells, when delivered via HAAM, exhibit enhanced potency, maintaining their pluripotency and viability [61]. This means that these cells can remain functional for a more extended period and have the capability to differentiate into a broader range of tissue types.

Cell viability, particularly in the context of HAAM, is crucial. In various studies, cells seeded onto HAAM have demonstrated prolonged viability, indicating that the membrane provides essential factors that promote cellular health and deter apoptosis [62]. The inherent properties of HAAM, such as its anti-inflammatory capabilities [63], contribute to creating a hospitable environment, ensuring that the transplanted cells are not only retained but also active and functional.

In essence, the use of HAAM as a cell delivery system allows for the direct delivery of therapeutic cells to the wound site, enhancing the body’s natural healing response and potentially improving overall outcomes in wound healing and tissue regeneration.

### 4.3. Unique Material Properties of HAAM: Biocompatibility

The unique material properties of HAAM, particularly its biocompatibility, play a crucial role in its application in tissue engineering and regenerative medicine. HAAM is derived from the amniotic membrane, a part of the placenta that naturally interfaces between the maternal and fetal circulatory systems. As such, it is a biological material that has inherently evolved to be “immune-privileged” or largely ignored by the immune system, contributing to its significant biocompatibility [64]. This immune privilege is largely due to HAAM’s unique composition, which includes a variety of proteins and growth factors that naturally reduce inflammation, fibrosis, and microbial growth. These include hyaluronic acid, collagens, laminin, fibronectin, and proteoglycans, all of which are critical components of the body’s ECM [64] and contribute to HAAM’s ability to promote wound healing.

The biodegradability of HAAM in vivo is another beneficial property for its use in tissue engineering [65]. This biodegradability means that the HAAM scaffold will naturally break down over time, reducing or eliminating the need for surgical removal once the wound has healed. The degradation rate of AM varies based on factors such as application or implantation site, the species in which it is used, and preservation methods [64]. This gradual degradation process also enables the continuous release of the HAAM’s inherent bioactive molecules over time, providing ongoing stimulation for tissue regeneration at the wound site.

The fluidity of HAAM, particularly when formulated into a hydrogel, provides another advantage in wound healing applications. Its ability to conform to the irregular shapes of wounds ensures optimal contact between the HAAM and the wound surface. This adaptability maximizes the exposure of the wound to the regenerative factors present in the HAAM, thereby enhancing the healing process [66].

In summary, while the comprehensive mechanisms underlying HAAM’s regenerative capabilities are yet to be fully unraveled, the current literature suggests a multifaceted process that encompasses the recruitment of healing cells, stimulation by growth factors, cell delivery, and unique material properties such as biocompatibility and adaptability to wound morphology. These properties make HAAM a promising material for applications in tissue engineering and regenerative medicine.

## 5. Preclinical Research on HAAM

Preclinical research on HAAM has provided valuable insights into its potential applications in regenerative medicine and tissue engineering. As summarized in Table 2, a series of preclinical studies have confirmed the research value of HAAM, providing a reference for its potential clinical applications.

### 5.1. Independent Applications

When applied alone, HAAM is commonly used as a skin dressing, surgical patch, and tissue engineering scaffold. For instance, Song et al. evaluated the effect of HAAM on promoting the healing of full-thickness skin defects in rats. They found that HAAM could promote the secretion of VEGF and α-SMA and reduce the expression of TGF-b1 in the early stage of recovery. Interestingly, the α-SMA level decreased in the HAAM group at day 14, significantly lower than in the control group. This indicates that the HAAM group reduced the angiogenesis of granulation tissue in the late stage of wound healing, thus reducing scar formation. Additionally, hair follicles were observed in the regenerated skin at around day 14. These findings indicate that HAAM could promote wound healing and skin appendage regeneration and also reduce wound inflammation and scar formation [15].

In addition, Gholipourmalekabadi et al. verified that HAAM showed strong antibacterial activity against *Escherichia coli* (ATCC 25922), *Staphylococcus aureus* (ATCC 25923), and *Pseudomonas aeruginosa* (ATCC 27853) isolated from burn patients but had no inhibitory effect on b-lactamase imipenemase-positive *P. aeruginosa* due to their high resistance to the tested antibiotics [67]. Cross-linking HAAM with a potent antimicrobial agent, such as antibiotics or silver ions, may solve this problem.

In addition to its application in wound repair, HAAM has been used as an abdominal patch [68], a pericardial patch [69], and a fetal membrane patch [70] and in bone/dental regeneration [71], hepatocyte differentiation [72], and as a 3D scaffold for in vitro tumor research [73]. These diverse applications highlight the versatility and potential of HAAM in the field of regenerative medicine and tissue engineering.

### 5.2. Combined Applications

The versatility of HAAM extends to its combined applications with other materials and cells, which have shown promising results in preclinical studies. For instance, Gholipourmalekabadi et al. prepared a 3D protein-based artificial skin made of HAAM and electrospun nanofiber silk fibroin which were loaded onto adipose tissue-derived mesenchymal stem cells and inserted into the third-degree full-thickness burn wounds of mice. The results showed accelerated neovascularization, early re-epithelialization, collagen tissue deposition, and the scarless healing of burn wounds [74]. In the same year, Kshersagar et al. compared the effects of synergistically activated HAAM’s platelet-rich plasma (PRP) with silver nitrate gel on burn wounds in mouse models. As a result, HAAM scaffolds activated with PRP showed well-differentiated epidermis, keratinocytes, hair follicles, and basement membrane hyperplasia, better promoting cell migration and skin regeneration. Furthermore, HAAM scaffolds activated with PRP and calcium chloride demonstrated better adhesion, keeping them attached in one place. HAAM requires only a single dressing, which minimizes the necessity of repeated dressings and decreases plasma exudation and fluid and heat loss from the operative area, which provides theoretical support for the clinical application of patients with extensive burns [17].

In addition, mesenchymal stem cells (MSCs) seeded on HAAM, such as placenta-derived MSCs and adipose–derived MSCs, have also been shown to promote the regeneration of skin appendages and have showed beneficial regenerative effects, including the highest rate of wound shrinkage, collagen production, and a significant reduction in inflammation [75]. Furthermore, HAAM-loaded MSCs have shown great potential for application in endometrial repair [18], muscle regeneration and differentiation [76], tracheal repair [77], epicardial repair [19], and in supporting neural differentiation [78].

**Table 2:** Preclinical research on HAAM.

Model	Number	Origin	Preparation Method	Sterilization Method	Group	Results	Reference
Full-thickness skin defects on the back of SD rats (2 month-old, male)	25	Healthy human placenta	1% Triton X-100 for 4 h, lipase (2000 U/L) for 10 h, and DNAase (2000 U/L) for 4 h at 37 °C.	Decellularization under the sterile state	Two groups: HAAM and control	HAAM increased the expression level of VEGF and α-SMA and decreased TGF-β1	[15]
In vitro experiment	N	Healthy human placenta	0.5 M NaOH for 30s, 0.2% EDTA for 30 min, cell scraper	N	Two groups: HAAM and fresh AM	HAAM had an antibacterial effect on three standard strains of ATCC bacteria	[67]
Myocardial infarction in Wistar rats (2–3 months old, male)	50	Healthy human placenta	0.01% SDS and 0.01% SD for 24 h at 37 °C	N	Three groups: HAAM, BMSC (bone-marrow mononuclear stem cells), control	HAAM has the potential for angiogenesis and cardiomyocyte regeneration	[69]
Iatrogenic defects in fetal membranes in a rabbit model.	8	Healthy human placenta	0.5% sodium deoxycholate, 0.02% ethylenediamine tetraacetic acid, two protease inhibitor cocktail tablets for 1 h at 4 °C, cell scraper	N	One group: HAAM	HAAM could restore the integrity of the punctured fetal membrane	[70]
In vitro experiment	N	Healthy human placenta	1.25% NaOCl for 5 min	70% ethanol for 3 h	Two groups: 3D HAAM scaffold and 2D cell culture model	HAAM could serve as a 3D scaffold for in vitro cancer research	[73]
In vitro osteogenic differentiation experiment	N	Healthy human placenta	0.1% EDTA for 2 h at 37 °C, cell scraper	N	One group: HAAM	HAAM promoted human APC (dental apical papilla cells) osteogenic differentiation	[71]
In vitro experiment	N	Healthy human placenta	Tris-EDTA over night in a refrigerator, SDS over night at 25 °C, Pepsin for 24 h at room temperature	N	One group: HAAM hiPSCs-HLCs (Human-induced pluripotent stem cells-derived hepatocyte-like cells)	HAAM supported the differentiation of hiPSCs to HLCs	[72]
In vitro experiment	N	Healthy human placenta	0.25% trypsin-EDTA for 20 min at 37 °C, cell scraper	N	One group: HAAM-e-CSF (Embryonic cerebrospinal fluid)-BM-MSCs (bone marrow derived mesenchymal stem cells)	HAAM could effectively improve BM-MSC cultivation and neural differentiation with e-CSF as a source of neurological factors	[78]
Second-degree burn injuries in balb/c mice (8–10 weeks old, male)	30	Healthy human placenta	acid peracetic	N	Two groups: HAAM and control	HAAM could promote the formation of vascularized granulation tissues and skin appendages while reducing the infiltration of inflammatory cells in the wound	[28]
Uterus of rabbits (1 year-old)	2	Healthy human placenta	1% Triton X-100 for 1 d, 2000 U/L lipase for 10 h, and 2000 U/L DNAase for 3 h	N	Two groups: HAAM/PU (poly(ester urethane) and PP (Polypropylene mesh)	HAAM/PU showed anti-inflammatory, high biocompatibility, and non-adherent to surrounding organs compared to the control group	[27]
Tendon injury models in chickens	30	Healthy human placenta	0.05% ethylenediaminetetraacetic acid at 37 °C for 2 h, cell scraper	ethylene oxide for 6 h	Two groups: HAAM and control	HAAM promoted the endogenous healing of the tendon and prevents exogenous adhesion	[54]
Burn wound dressing of mouse (6 month-old female)	3	Healthy human placenta	2% sodium deoxycholeate (SD) (*w*/*v*) for 6 h; 2% SDS for 6 h	N	Three groups: HAAM synergistically activated PRP, silver nitrate gel, control	HAAM synergistically activated PRP-accelerated cell migration and skin regeneration compared to the other two groups	[17]
Abdominal defect in SD rats	20	Healthy human placenta	1% TritonX-100 for 24 h; 0.25% trypsin and 0.02% EDTA for 4 h at 37 °C	γ-rays (30 kGy)	Two groups: electrospun HAAM and SIS mesh (small intestinal submucosa)	Electrospun HAAM showed superior bioactivity and reinforced mechanical support	[68]
Third-degree burn injuries in BALB/c mouse (male)	75	Healthy human placenta	N	N	Five groups: HAAM/ESF/AT-MSCs, HAAM/ESF, HAAM/AT-MSCs, HAAM, control	HAAM and other experimental groups showed accelerated wound healing, neo-vascularization, and early re-epithelialization compared to the control group (*p* < 0.005)	[74]
Excisional wound in Wistar rats (9–10 week-old, male)	24	Healthy human placenta	0.05% Trypsin-EDTA for 30 min at 37 °C	N	Four groups: HAAM-PLMSCs (placenta-derived mesenchymal stem cells), HAAM-ADMSCs (adipose–derived mesenchymal stem cells), HAAM, control	HAAM and other experimental groups showed accelerated wound healing and regeneration of skin appendages compared to the control	[75]
Endometrial injury in SD rats (6–8 weeks old, female)	48	Healthy human placenta	0.1% Triton X-100 for 36 h at 37 °C; 2.5% trypsin-EDTA for 4 h at 37 °C, cell scraper	N	Four groups: HAAM-UCMSCs (Umbilicalcord-derived mesenchymal stem cells), HAAM, normal, control	The HAAM-UCMSCs group could promote the proliferation of endometrial epithelial and stromal cells	[18]
In vitro experiment	N	Healthy human placenta	Three freeze–thaw; trypsin-EDTA overnight at 4 °C, cell scraper	N	One group: HAAM-PCL (Poly(ε-caprolactone))	HAAM-PCL facilitated the myogenic differentiation of ADSCs	[76]
Tracheal defects in New Zealand rabbits	30	Healthy human placenta	0.01% SDS and 0.01% SD for 24 h at 37 °C	N	Three groups: HAAM-hucMSCs (human umbilical cord mesenchymal stem cells), HAAM, control	HAAM facilitated hucMSCs differentiated into chondrocytes	[77]
In vitro experiment	N	Healthy human placenta	10 mM Tris and 0.1% EDTA for 1 h; 0.5% SDS for 4 h at room temperature	N	One group: HAAM-human cardiac ECM hydrogel	HAAM-human cardiac ECM hydrogel could specifically support the culture and interaction of cardiac cells	[19]
In vitro experiment and in vivo experiment (full-thickness skin defects in New Zealand rabbits, male and female)	72	Healthy human placenta	N	N	One group (in vitro experiment): GelMA-dHAMMA composite hydrogel (methacrylated gelatin (GelMA), dHAM-methacrylic anhydride(dHAMMA)); Three groups: (vivo experiment): -GelMA-dHAMMA -GelMA -control	GelMA-dHAMMA could promote fibroblast proliferation and α-SMA expression in an in vitro experiment and promote wound healing in an in vivo experiment	[60]
Full-thickness skin defects in New Zealand rabbits (6 months old)	24	Healthy rabbit placenta	1% Triton X-100 for 12 h; 0.25% trypsin and 0.02% EDTA for 1 h, and RNaseA (0.02 mg/mL) and DNaseI(0.2 mg/mL) for 4 h at 37 °C	γ-rays	Four groups: AM (HAAM), PAM (polyacrylamide), AlgSr/PAM (Polyacrylamide-alginate gel), AlgSrIII/PAM-AM	AlgSrIII/PAM-AM could effectively promote the endothelialization process and repair blood vessels	[66]
Third-degree burn wound in New Zealand white rabbits (adult female/male)	4	Healthy human placenta	1% Triton X-100 and 1% SDC at 4 °C for 48 h	plasma (H_2_O_2_) at 48 °C	Four groups: AMFIBHA (acellular amniotic membrane(AM), fibrin (FIB), hyaluronic acid (HA)); cpAM (cellular and plasma sterilized AM); pdAM (plasma sterilization dAM); control	AMFIBHA group could promote complete the epithelialization of the wound compared to the other groups and showed good stability	[58]

N represents Not Mentioned.

**Figure 2:**
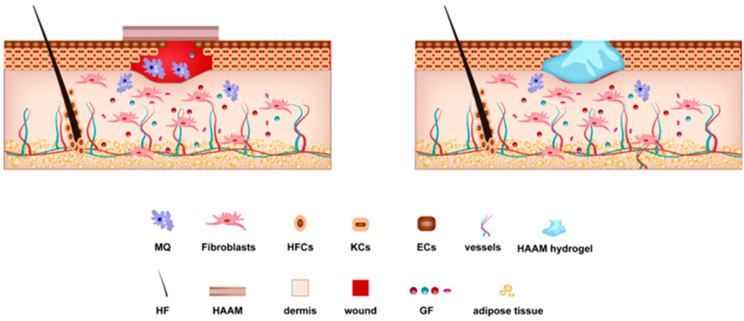
Mechanism through which the HAAM and HAAM hydrogel promote wound healing.

HAAM can also be prepared as hydrogels for application to skin wounds [60]. For example, Lei et al. prepared a composite hydrogel with high elasticity, high mechanical stability, high bioactivity, and a low swelling rate after cross-linking a HAAM hydrogel and polyacrylamide sodium alginate gel, which compensated for the weak mechanical strength and tensile strength of HAAM [66]. The hydrogel provided good adhesion to the skin, and this was matched with good physical properties such as flexibility and elasticity, so that it could rapidly gel and fill the wound area to exert effects regardless of the size or shape of the wound [79]. Figure 2 shows the mechanism through which the HAAM and HAAM hydrogel promote wound healing.

Both the independent and combined applications of HAAM have shown promising results in preclinical studies, highlighting its potential for use in regenerative medicine and tissue engineering applications.

## 6. Clinical Applications of HAAM

Recently, HAAM has also been gradually applied clinically with the vigorous development of tissue engineering. As shown in Table 3, HAAM has been used independently as a dressing and in a combinational sense as a scaffold, which fully demonstrates its safety and effectiveness.

### 6.1. Independent Applications

The independent applications of HAAM have been explored in various clinical settings, demonstrating its safety and effectiveness in promoting healing. Nouri et al. compared the healing effects of HAAM and Mepitel on the regeneration of the donor region of STSG. The results showed that HAAM’s re-epithelization results, pain sensation during dressing change, and Vancouver scar score (3 and 6 months post operation) were similar to those of Mepitel, confirming the safety and effectiveness of HAAM in promoting STSG donor site healing [80]. Another clinical trial confirmed that HAAM improved wound healing faster than silver sulfadiazine gauze treatment among 12 patients with second-degree burn wounds, and there were no signs of infection, immunologic reaction, or dermatitis in any wound treated with HAAM [28].

In addition, HAAM has been used for treating venous lower-limb ulcers with no clear exudation of the ulcer; in one study pain score was reduced significantly after 1 week of treatment, with no local inflammatory or allergic reactions, and ease of management enabled patients to dress their own wounds after discharge, which reduced medical costs and helped to ensure patient compliance [81]. Moreover, in a clinical study regarding lower-third nasal reconstruction via HAAM transplantation, the HAAM treatment group showed significantly reduced hemostasis times (*p* < 0.001), wound infection, and scar formation (*p* < 0.05) and an accelerated disappearance of postoperative pain (*p* < 0.001) and postoperative scab formation and detachment (*p* < 0.001) [82].

Palaniappan Ramasamy et al. applied wet-HAAM and dry-HAAM for conjunctival reconstruction after the surgical removal of the nasal pterygium. Twenty-one days after surgery, the development of functional epithelium on the conjunctival surface was complete, and the HAAM appeared to dissolve and disappear. One month later, the wound in the operative area was completely healed [16]. Additionally, the full-thickness auricular skin defects [20] and bone/dental regeneration [83] verified the above conclusions, meaning that HAAM is an ideal biological dressing for facial wounds.

### 6.2. Combined Applications

The combined applications of HAAM, particularly in conjunction with other cells, have shown promising results in various clinical settings. Hashemi et al. transplanted HAAM-loaded dermal fibroblasts and Wharton’s jelly mesenchymal stem cells into the chronic wounds of patients with diabetes. This study demonstrated surprising effects as HAAM promoted wound healing with complete skin regeneration and re-epithelialization after just 9 days of treatment, with no side effects or complications [84]. In the study, the proposed cell therapy technique enabled wounds that previously took over a year to heal to be healed in less than two weeks, supporting the efficacy of new therapeutic approaches for managing chronic and difficult-to-heal wounds using skin tissue engineering and regenerative medicine.

In a previous preliminary study, Hashemi et al. transplanted HAAM-loaded umbilical cord Wharton’s jelly stem cells to treat diabetic foot ulcers. The wound decreased significantly after 6 and 9 days (*p* < 0.002), providing further support for the effectiveness of HAAM in wound healing [85]. HAAM acts as a biological scaffold and loads cells, and these cells can be involved in coordinating the healing process through intercellular interactions as well as autocrine and paracrine signaling, resulting in accelerated wound healing.

In conclusion, as Figure 2 shows, HAAM has shown potential in preclinical research and clinical applications, such as skin transplantation and wound healing, surgical patches, cell culture matrix and cell delivery system application, etc. Therefore, HAAM is a very promising skin dressing and biological scaffold which will play a crucial role in the future of tissue engineering and reconstructive surgery.

**Table 3:** Clinical applications of HAAM.

Defects	Year	Preparation Method	Sterilization Method	Study Design	Number of Patients	Results	Follow-up (Months)	Trial Registration Number	Reference
Split-thickness graft donor site	2018	EDTA (0.025%) for 1 h and cell scraper	γ-rays (25 kGy)	Three groups: Mepitel, Dried AM, HAAM	20	HAAM has no significant differences with Mepitel, including re-epithelization, pain sensation, scar formation, and infection rate.	6	IRCT201511118177N12	[80]
Second-degree-burned skin	2019	acid peracetic	N	Two groups: HAAM and silver sulfadiazine gauze	12	HAAM showed accelerated wound healing compared to the control group (*p* < 0.001).	N	N	[28]
Venous lower-limb ulcers	2013	glutaraldehyde, 0.5% SDS for 24 h at 4 °C, 0.25% trypsin for 4 h at 37 °C	ethylene oxide	One group: HAAM	4	HAAM showed accelerated wound healing and generated less pain and lower medical costs.	6	N	[81]
Full-thickness defects in the lower third of the nose	2012–2016	SDS for 5 h; 0.25% trypsin for 6 h	γ-rays	Two groups: HAAM and Vaseline gauze	180	HAAM showed accelerated wound healing and fewer complications (*p* < 0.001).	3	ChiCTR1800017618	[82]
Full- thickness auricular skin defects after benign tumor removal	2016–2021	N	N	Two groups: HAAM and Vaseline gauze	36	HAAM showed accelerated wound healing and fewer complications compared to the control group (*p* < 0.05).	3	N	[20]
Chronic diabetic foot ulcers	2020	0.25% Trypsin- EDTA, mechanical isolation	N	One group: -HAAM-loaded WJ-MSCs and DFs	5	HAAM-loaded WJ-MSCs and DFs showed accelerated wound healing and no side effects or complications.	1	N	[84]
Chronic diabetic wounds	2019	N	N	One group: HAAM-loaded WJ-MSCs	5	HAAM-loaded WJ-MSCs showed accelerated wound healing (*p* < 0.002).	1	N	[85]
Ocular surface diseases	2021	EDTA (0.25% *w*/*v*)	γ-rays (25 kGy)	Two groups: PW-HAAM (processed wet HAAM) and PD-HAAM (processed dry HAAM)	N	HAAM repaired the conjunctival surface.	N	N	[16]

N represents Not Mentioned.

## 7. Discussion

### 7.1. Potential of HAAM in the Treatment of Extensive and Deep Skin Damage

The potential of HAAM in the treatment of extensive and deep skin damage, a significant challenge in dermatology and plastic surgery, is promising. Currently, autologous skin transplantation is the optimal treatment for skin injuries and burns. However, this method has limitations, including insufficient donor sites, damage to donor sites, and the possibility of transplantation failure. Other alternative treatments, such as allogeneic skin grafting and the application of advanced skin dressings, have been limited in their widespread use due to immune rejection, poor skin quality, high cost, and scar formation. With the emergence and progress of biotechnology and tissue engineering technology, skin substitutes have become an effective tool for the treatment of skin wounds and have been actively studied for their contribution to the management of chronic wounds [86].

However, several challenges and unresolved questions need to be addressed to fully harness its potential. One of the primary challenges lies in the efficiency of the decellularization and sterilization methods. Some methods, while effective, can easily destroy the ECM and leave behind chemical reagents [14]. This could potentially affect the integrity of the HAAM and its subsequent performance in clinical applications. Therefore, there is a need for more efficient methods that can ensure complete decellularization without compromising the ECM. HAAMs can be kept at room temperature for 3–5 years and are easily transportable and available year-round. In this review, we described three methods for preparing HAAM and four methods for sterilizing HAAM. Among them, the chemical decellularization method has low efficiency and can easily destroy the ECM [14]. The biological method has high acellularization efficiency but high costs, and enzyme concentrations need to be accurately controlled to avoid damaging the biomechanical properties of HAAM [34]. Physical decellularization is often used as an auxiliary method due to its gentleness. Therefore, the acellular method is often used in combination with other methods to fully prepare HAAM with high efficiency.

In addition, sterilization minimizes the risk of disease transmission by HAAM. However, some sterilization methods, such as irradiation, can affect the ECM’s structure [43]. Ethylene oxide, while effective, has the disadvantages of being flammable, explosive, and toxic [44]. Peracetic acid can be used for both the decellularization and sterilization of HAAM but may interfere with tissue structures [43]. SC-CO_2_ overcomes the limitations of conventional sterilization by eliminating biological contaminants while retaining tissue bioactivity [46].

### 7.2. Challenges in HAAM Use and Future Directions

HAAM has shown significant promise in the field of regenerative medicine, particularly in wound healing and tissue regeneration. However, several challenges and limitations need to be addressed to fully harness its potential.

- Mechanism of Action Studies: More in-depth studies are needed to understand the role of HAAM’s ECM components, like collagen types III, IV, and V, proteoglycans, and glycoproteins, in cell adhesion, growth, and differentiation. Gaining a deeper understanding of how these components affect wound healing at the molecular level can lead to better application strategies;
- Customizing HAAM for Specific Applications: HAAM’s versatility as a surgical patch, tissue scaffold, and a cell delivery vehicle has been highlighted. However, different medical applications might have distinct requirements. Research can be directed towards customizing HAAM for specific uses, such as exploring its ability to deliver specific types of drugs, cells, or growth factors for targeted treatments;
- Mitigating Disease Transmission Risks: While the decellularization of the amniotic membrane reduces immunogenicity, potential risks of disease transmission remain. Studies to establish more robust sterilization processes or assess the risk profile for disease transmission would be beneficial;
- Integration with Other Therapies: As an ideal skin substitute and biological scaffold, HAAM’s compatibility with other therapeutic strategies, e.g., gene therapy, stem cell therapy, or nanoparticle delivery systems, could be further explored.

By focusing on these specific areas, the use of HAAM in wound care and regenerative medicine can be further optimized, paving the way for more efficient, cost-effective, and safer methods for the decellularization and sterilization of HAAM while ensuring the retention of tissue bioactivity.

## 8. Conclusions

Our review has clarified the profound potential of HAAM in advancing regenerative medicine, specifically with respect to its use as a skin substitute and biological scaffold. To fully harness its potential, it is critical to address the challenges and limitations associated with its production, preservation, sterilization, and transportation.

Improved preparation and application methods that are cost-effective and maximize the potential advantages of HAAM in various applications should be the focus of future research. SC-CO_2_ has shown promise as a decellularization method and sterilization agent, preserving the extracellular matrix structure and maintaining biological properties while being non-toxic and environmentally friendly [45,87]. However, the low mechanical strength of HAAM has limited its practical application. Current solutions include stacking layers of HAAM [88], preparing composite materials [76], creating HAAM hydrogels [66], or crosslinking [35]. The incorporation of functional groups into HAAM is also an effective method for improving the performance of the scaffold [89]. The use of multiple or multifunctional scaffolds that can achieve enhanced synergy when combined will undoubtedly play a greater role than a single scaffolds [58]. However, the potential mechanism underlying HAAM’s regeneration ability is still unclear and requires further research. Moreover, while HAAM has shown its safety and effectiveness in preclinical and clinical research, it is unethical and unacceptable to perform biopsies on healing the skin of patients. Therefore, a randomized, multicenter, double-blind, and controlled trial is required to further test the safety and effectiveness of HAAM implantation in clinical settings.

In conclusion, HAAM presents a promising solution for wound healing and tissue regeneration; however, these potential future directions highlight the need for ongoing research and developments to fully realize its potential. To overcome the current limitations and open new avenues for HAAM application, a multi-disciplinary approach that combines insights from tissue engineering, cell biology, and clinical medicine is required.

## Data Availability

Not applicable.

## Abbreviations

HAAM	Human acellular amniotic membrane
SC-CO_2_	Supercritical Carbon Dioxide
WHO	World Health Organization
STSG	split thickness skin grafting
AM	Amnion membrane
ECM	extracellular matrix
SDS	sodium dodecyl sulfate
HAM	Human Amniotic Membrane
EDTA	ethylenediaminetetraacetic acid
TGF-β	Transforming Growth Factor-beta
EGF	epidermal growth factor
VEGF	vascular endothelial growth factor
FGF	fibroblast growth factor
PRP	platelet rich plasma
MSCs	mesenchymal stem cells
NTIRE	nonthermal irreversible electroporation

## References

[1] Tiwari N., Kumar D., Priyadarshani A., Jain G.K., Mittal G., Kesharwani P., Aggarwal G. (2023). Recent progress in polymeric biomaterials and their potential applications in skin regeneration and wound care management. J. Drug Deliv. Sci. Technol..

[2] Widjaja W., Tan J., Maitz P.K.M. (2017). Efficacy of dermal substitute on deep dermal to full thickness burn injury: A systematic review. ANZ J. Surg..

[3] Goyer B., Larouche D., Kim D.H., Veillette N., Pruneau V., Bernier V., Auger F.A., Germain L. (2019). Immune tolerance of tissue-engineered skin produced with allogeneic or xenogeneic fibroblasts and syngeneic keratinocytes grafted on mice. Acta Biomater..

[4] Climov M., Medeiros E., Farkash E.A., Qiao J., Rousseau C.F., Dong S., Zawadzka A., Racki W.J., Al-Musa A., Sachs D.H. (2016). Bioengineered Self-assembled Skin as an Alternative to Skin Grafts. Plastic and reconstructive surgery. Glob. Open.

[5] Fishman J.A. (2020). Prevention of infection in xenotransplantation: Designated pathogen-free swine in the safety equation. Xenotransplantation.

[6] Savoji H., Godau B., Hassani M.S., Akbari M. (2018). Skin Tissue Substitutes and Biomaterial Risk Assessment and Testing. Front. Bioeng. Biotechnol..

[7] Halim A.S., Khoo T.L., Yussof S.J.M. (2010). Biologic and synthetic skin substitutes: An overview. Indian J. Plast. Surg..

[8] Loeffelbein D.J., Baumann C., Stoeckelhuber M., Hasler R., Mucke T., Steinstrasser L., Drecoll E., Wolff K.D., Kesting M.R. (2012). Amniotic membrane as part of a skin substitute for full-thickness wounds: An experimental evaluation in a porcine model. J. Biomed. Mater. Res. Part B Appl. Biomater..

[9] Mohammadi A.A., Jafari S.M.S., Kiasat M., Tavakkolian A.R., Imani M.T., Ayaz M., Tolide-ie H.R. (2013). Effect of fresh human amniotic membrane dressing on graft take in patients with chronic burn wounds compared with conventional methods. Burns.

[10] Ditmars F.S., Lind R.A., Broderick T.C., Fagg W.S. (2022). Safety and efficacy of acellular human amniotic fluid and membrane in the treatment of non-healing wounds in a patient with chronic venous insufficiency. SAGE Open Med. Case Rep..

[11] Riau A.K., Beuerman R.W., Lim L.S., Mehta J.S. (2010). Preservation, sterilization and de-epithelialization of human amniotic membrane for use in ocular surface reconstruction. Biomaterials.

[12] Mohan R., Bajaj A., Gundappa M. (2017). Human Amnion Membrane: Potential Applications in Oral and Periodontal Field. J. Int. Soc. Prev. Community Dent..

[13] Filipas D. (2001). Vaginal reconstruction/fistulae. Curr. Opin. Urol..

[14] Wilshaw S.P., Kearney J., Fisher J., Ingham E. (2008). Biocompatibility and potential of acellular human amniotic membrane to support the attachment and proliferation of allogeneic cells. Tissue Eng. Part A.

[15] Song M., Wang W., Ye Q., Bu S., Shen Z., Zhu Y. (2017). The repairing of full-thickness skin deficiency and its biological mechanism using decellularized human amniotic membrane as the wound dressing. Mater. Sci. Eng. C Mater. Biol. Appl..

[16] Ramasamy P., Krishnakumar R., Rekha R., Vaseeharan B., Saraswathi K., Raj M., Hanna R.E.B., Brennan G.P., Dayanithi G., Vijayakumar S. (2021). Bio-Fabrication of Human Amniotic Membrane Zinc Oxide Nanoparticles and the Wet/Dry HAM Dressing Membrane for Wound Healing. Front. Bioeng. Biotechnol..

[17] Kshersagar J., Kshirsagar R., Desai S., Bohara R., Joshi M. (2018). Decellularized amnion scaffold with activated PRP: A new paradigm dressing material for burn wound healing. Cell Tissue Bank..

[18] Wang S., Shi C., Cai X., Wang Y., Chen X., Han H., Shen H. (2021). Human Acellular Amniotic Matrix with Previously Seeded Umbilical Cord Mesenchymal Stem Cells Restores Endometrial Function in a Rat Model of Injury. Mediat. Inflamm..

[19] Becker M., Maring J.A., Schneider M., Martin A.X.H., Seifert M., Klein O., Braun T., Falk V., Stamm C. (2018). Towards a Novel Patch Material for Cardiac Applications: Tissue-Specific Extracellular Matrix Introduces Essential Key Features to Decellularized Amniotic Membrane. Int. J. Mol. Sci..

[20] Chen Y., Lyu L., Xue S. (2021). Evaluation of human acellular amniotic membrane for promoting anterior auricle reconstruction. Exp. Dermatol..

[21] Zhou H., Wang L., Zhang C., Hu J., Chen J., Du W., Liu F., Ren W., Wang J., Quan R. (2019). Feasibility of repairing full-thickness skin defects by iPSC-derived epithelial stem cells seeded on a human acellular amniotic membrane. Stem Cell Res. Ther..

[22] Arrizabalaga J., Nollert M.U. (2018). Human Amniotic Membrane: A Versatile Scaffold for Tissue Engineering. ACS Biomater. Sci. Eng..

[23] Cui H., Chai Y., Yu Y. (2019). Progress in developing decellularized bioscaffolds for enhancing skin construction. J. Biomed. Mater. Res. Part A.

[24] Sanluis-Verdes A., Vilar M.Y.-P., García-Barreiro J., García-Camba M., Ibáñez J., Doménech N., Rendal-Vázquez M.E. (2015). Production of an acellular matrix from amniotic membrane for the synthesis of a human skin equivalent. Cell Tissue Bank..

[25] Gholipourmalekabadi M., Mozafari M., Salehi M., Seifalian A., Bandehpour M., Ghanbarian H., Urbanska A.M., Sameni M., Samadikuchaksaraei A., Seifalian A.M. (2015). Development of a Cost-Effective and Simple Protocol for Decellularization and Preservation of Human Amniotic Membrane as a Soft Tissue Replacement and Delivery System for Bone Marrow Stromal Cells. Adv. Healthc. Mater..

[26] Xing Q., Yates K., Tahtinen M., Shearier E., Qian Z., Zhao F. (2015). Decellularization of fibroblast cell sheets for natural extracellular matrix scaffold preparation. Tissue Eng. Part C Methods.

[27] Shi P., Gao M., Shen Q., Hou L., Zhu Y., Wang J. (2015). Biocompatible surgical meshes based on decellularized human amniotic membrane. Mater. Sci. Eng. C Mater. Biol. Appl..

[28] Milan P.B., Amini N., Joghataei M.T., Ebrahimi L., Amoupour M., Sarveazad A., Kargozar S., Mozafari M. (2020). Decellularized human amniotic membrane: From animal models to clinical trials. Methods.

[29] Gilbert T.W., Sellaro T.L., Badylak S.F. (2006). Decellularization of tissues and organs. Biomaterials.

[30] Ashouri S., Hosseini S.A., Hoseini S.J., Tara F., Ebrahimzadeh-Bideskan A., Webster T.J., Kargozar S. (2022). Decellularization of human amniotic membrane using detergent-free methods: Possibilities in tissue engineering. Tissue Cell.

[31] Khosravimelal S., Momeni M., Gholipur M., Kundu S.C., Gholipourmalekabadi M. (2020). Protocols for decellularization of human amniotic membrane. Methods Cell Biol..

[32] Guo Q., Lu X., Xue Y., Zheng H., Zhao X., Zhao H. (2012). A new candidate substrate for cell-matrix adhesion study: The acellular human amniotic matrix. J. Biomed. Biotechnol..

[33] Chen C., Zheng S., Zhang X., Dai P., Gao Y., Nan L., Zhang Y., Zhao F., Zhou L., Xu Z. (2018). Transplantation of Amniotic Scaffold-Seeded Mesenchymal Stem Cells and/or Endothelial Progenitor Cells from Bone Marrow to Efficiently Repair 3-cm Circumferential Urethral Defect in Model Dogs. Tissue Eng. Part A.

[34] Fenelon M., Maurel D.B., Siadous R., Gremare A., Delmond S., Durand M., Brun S., Catros S., Gindraux F., L’Heureux N. (2019). Comparison of the impact of preservation methods on amniotic membrane properties for tissue engineering applications. Mater. Sci. Eng. C Mater. Biol. Appl..

[35] Huang G., Ji S., Luo P., Liu H., Zhu S., Wang G., Zhou P., Xiao S., Xia Z. (2013). Accelerated expansion of epidermal keratinocyte and improved dermal reconstruction achieved by engineered amniotic membrane. Cell Transplant..

[36] Mahmoudi-Rad M., Abolhasani E., Moravvej H., Mahmoudi-Rad N., Mirdamadi Y. (2013). Acellular amniotic membrane: An appropriate scaffold for fibroblast proliferation. Clin. Exp. Dermatol..

[37] Pulver, Shevtsov A., Leybovich B., Artyuhov I., Maleev Y., Peregudov A. (2014). Production of organ extracellular matrix using a freeze-thaw cycle employing extracellular cryoprotectants. Cryoletters.

[38] Crapo P.M., Gilbert T.W., Badylak S.F. (2011). An overview of tissue and whole organ decellularization processes. Biomaterials.

[39] Phillips M., Maor E., Rubinsky B. (2010). Nonthermal irreversible electroporation for tissue decellularization. J. Biomech. Eng..

[40] Zemmyo D., Yamamoto M., Miyata S. (2021). Efficient Decellularization by Application of Moderate High Hydrostatic Pressure with Supercooling Pretreatment. Micromachines.

[41] Bujang-Safawi E., Halim A.S., Khoo T.L., Dorai A.A. (2010). Dried irradiated human amniotic membrane as a biological dressing for facial burns—A 7-year case series. Burns.

[42] Singh R., Purohit S., Chacharkar M.P., Bhandari P.S., Bath A.S. (2007). Microbiological safety and clinical efficacy of radiation sterilized amniotic membranes for treatment of second-degree burns. Burns.

[43] von Versen-Höynck F., Syring C., Bachmann S., Möller D.E. (2004). The influence of different preservation and sterilisation steps on the histological properties of amnion allografts—Light and scanning electron microscopic studies. Cell Tissue Bank..

[44] Xiao S., Xiao C., Miao Y., Wang J., Chen R., Fan Z., Hu Z. (2021). Human acellular amniotic membrane incorporating exosomes from adipose-derived mesenchymal stem cells promotes diabetic wound healing. Stem Cell Res. Ther..

[45] Chou P., Lin Y., Wu S., Lin S., Srinivasan P., Hsieh D., Huang S.H. (2020). Supercritical Carbon Dioxide-decellularized Porcine Acellular Dermal Matrix combined with Autologous Adipose-derived Stem Cells: Its Role in Accelerated Diabetic Wound Healing. Int. J. Med. Sci..

[46] Wehmeyer J.L., Natesan S., Christy R.J. (2015). Development of a Sterile Amniotic Membrane Tissue Graft Using Supercritical Carbon Dioxide. Tissue Eng. Part C Methods.

[47] Chen X., Zhou Y. (2018). Preventive effects of transplantation of oral mucosal epithelial cells seeded on a decellularized amniotic membrane in a model of intrauterine adhesion. Int. J. Clin. Exp. Pathol..

[48] Tang K., Wu J., Xiong Z., Ji Y., Sun T., Guo X. (2018). Human acellular amniotic membrane: A potential osteoinductive biomaterial for bone regeneration. J. Biomater. Appl..

[49] Francisco J.C., Cunha R.C., Cardoso M.A., Simeoni R.B., Mogharbel B.F., Picharski G.L., Dziedzic D.S.M., Guarita-Souza L.C., Carvalho K.A.T. (2016). Decellularized amniotic membrane scaffold as a pericardial substitute: An in vivo study. Transplant. Proc..

[50] Salah R.A., Mohamed I.K., El-Badri N. (2018). Development of decellularized amniotic membrane as a bioscaffold for bone marrow-derived mesenchymal stem cells: Ultrastructural study. J. Mol. Histol..

[51] Han X., Ma Y., Lu X., Li W., Xia E., Li T.-C., Zhang H., Huang X. (2020). Transplantation of human adipose stem cells using acellular human amniotic membrane improves angiogenesis in injured endometrial tissue in a rat intrauterine adhesion model. Cell Transplant..

[52] Kim S.Y., Nair M.G. (2019). Macrophages in wound healing: Activation and plasticity. Immunol. Cell Biol..

[53] Bainbridge P. (2013). Wound healing and the role of fibroblasts. J. Wound Care.

[54] Sang R., Liu Y., Kong L., Qian L., Liu C. (2020). Effect of Acellular Amnion with Increased TGF-β and bFGF Levels on the Biological Behavior of Tenocytes. Front. Bioeng. Biotechnol..

[55] Tracy L.E., Minasian R.A., Caterson E.J. (2016). Extracellular Matrix and Dermal Fibroblast Function in the Healing Wound. Adv. Wound Care.

[56] Mathew-Steiner S.S., Roy S., Sen C.K. (2021). Collagen in Wound Healing. Bioengineering.

[57] Laleh M., Tahernejad M., Bonakdar S., Asefnejad A., Golkar M., Kazemi-Lomedasht F., Habibi-Anbouhi M. (2023). Positive effect of acellular amniotic membrane dressing with immobilized growth factors in skin wound healing. J. Biomed. Mater. Res. Part A.

[58] Ramakrishnan R., Sreelatha H., Anil A., Arumugham S., Varkey P., Senan M., Krishnan L.K. (2020). Human-Derived Scaffold Components and Stem Cells Creating Immunocompatible Dermal Tissue Ensuing Regulated Nonfibrotic Cellular Phenotypes. ACS Biomater. Sci. Eng..

[59] Gholipourmalekabadi M., Sameni M., Radenkovic D., Mozafari M., Mossahebi-Mohammadi M., Seifalian A. (2016). Decellularized human amniotic membrane: How viable is it as a delivery system for human adipose tissue-derived stromal cells?. Cell Prolif..

[60] Zhang Q., Chang C., Qian C., Xiao W., Zhu H., Guo J., Meng Z., Cui W., Ge Z. (2021). Photo-crosslinkable amniotic membrane hydrogel for skin defect healing. Acta Biomater..

[61] Parolini O., Soncini M., Evangelista M., Schmidt D. (2009). Amniotic membrane and amniotic fluid-derived cells: Potential tools for regenerative medicine?. Regen. Med..

[62] Moravvej H., Memariani H., Memariani M., Kabir-Salmani M., Shoae-Hassani A., Abdollahimajd F. (2021). Evaluation of fibroblast viability seeded on acellular human amniotic membrane. BioMed Res. Int..

[63] Francisco J.C., Uemura L., Simeoni R.B., da Cunha R.C., Mogharbel B.F., Simeoni P.R.B., Naves G., Napimoga M.H., Noronha L., Carvalho K.A.T. (2020). Acellular human amniotic membrane scaffold with 15d-PGJ2 nanoparticles in postinfarct rat model. Tissue Eng. Part A.

[64] Fénelon M., Catros S., Meyer C., Fricain J.C., Obert L., Auber F., Louvrier A., Gindraux F. (2021). Applications of Human Amniotic Membrane for Tissue Engineering. Membranes.

[65] Bhawna, Gujjar S., Venkataprasanna K.S., Tiwari S., Sharma J.C., Sharma P., Pujani M., Pandey A.K., Abnave P., Kalyanasundaram D. (2023). Stabilized human amniotic membrane for enhanced sustainability and biocompatibility. Process Biochem..

[66] Lei X., Wu Y., Peng X., Zhao Y., Zhou X., Yu X. (2020). Research on alginate-polyacrylamide enhanced amnion hydrogel, a potential vascular substitute material. Mater. Sci. Eng. C Mater. Biol. Appl..

[67] Gholipourmalekabadi M., Bandehpour M., Mozafari M., Hashemi A., Ghanbarian H., Sameni M., Salimi M., Gholami M., Samadikuchaksaraei A. (2015). Decellularized human amniotic membrane: More is needed for an efficient dressing for protection of burns against antibiotic-resistant bacteria isolated from burn patients. Burns.

[68] Liu Z., Zhu X., Zhu T., Tang R. (2020). Evaluation of a Biocomposite Mesh Modified with Decellularized Human Amniotic Membrane for Intraperitoneal Onlay Mesh Repair. ACS Omega.

[69] Blume G.G., Machado-Junior P.A.B., Simeoni R.B., Bertinato G.P., Tonial M.S., Nagashima S., Pinho R.A., de Noronha L., Olandoski M., de Carvalho K.A.T. (2021). Bone-Marrow Stem Cells and Acellular Human Amniotic Membrane in a Rat Model of Heart Failure. Life.

[70] Mallik A.S., Fichter M.A., Rieder S., Bilic G., Stergioula S., Henke J., Schneider K.T., Kurmanavicius J., Biemer E., Zimmermann R. (2007). Fetoscopic closure of punctured fetal membranes with acellular human amnion plugs in a rabbit model. Obstet. Gynecol..

[71] Chen Y.J., Chung M.C., Yao C.C.J., Huang C.H., Chang H.H., Jeng J.H., Young T.H. (2012). The effects of acellular amniotic membrane matrix on osteogenic differentiation and ERK1/2 signaling in human dental apical papilla cells. Biomaterials.

[72] Abazari M.F., Soleimanifar F., Enderami S.E., Nasiri N., Nejati F., Mousavi S.A., Soleimani M., Kiani J., Ghoraeian P., Kehtari M. (2020). Decellularized amniotic membrane Scaffolds improve differentiation of iPSCs to functional hepatocyte-like cells. J. Cell. Biochem..

[73] Ganjibakhsh M., Mehraein F., Koruji M., Aflatoonian R., Farzaneh P. (2019). Three-dimensional decellularized amnion membrane scaffold as a novel tool for cancer research; cell behavior, drug resistance and cancer stem cell content. Mater. Sci. Eng. C Mater. Biol. Appl..

[74] Gholipourmalekabadi M., Seifalian A.M., Urbanska A.M., Omrani M.D., Hardy J.G., Madjd Z., Hashemi S.M., Ghanbarian H., Milan P.B., Mozafari M. (2018). 3D Protein-Based Bilayer Artificial Skin for the Guided Scarless Healing of Third-Degree Burn Wounds in Vivo. Biomacromolecules.

[75] Aghayan H.R., Hosseini M.S., Gholami M., Mohamadi-Jahani F., Tayanloo-Beik A., Alavi-Moghadam S., Payab M., Goodarzi P., Abdollahi M., Larijani B. (2022). Mesenchymal stem cells’ seeded amniotic membrane as a tissue-engineered dressing for wound healing. Drug Deliv. Transl. Res..

[76] Hadipour A., Bayati V., Rashno M., Orazizadeh M. (2021). Aligned Poly(epsilon-caprolactone) Nanofibers Superimposed on Decellularized Human Amniotic Membrane Promoted Myogenic Differentiation of Adipose Derived Stem Cells. Cell J..

[77] Simeoni P.R.B., Simeoni R.B., Junior P.A.B.M., de Almeida M.B., Dziedzic D.S.M., da Rosa N.N., Stricker P.E.F., Miggiolaro A.F.R.D.S., Naves G., Neto N.B. (2021). Tracheal Repair with Human Umbilical Cord Mesenchymal Stem Cells Differentiated in Chondrocytes Grown on an Acellular Amniotic Membrane: A Pre-Clinical Approach. Life.

[78] Dorazehi F., Nabiuni M., Jalali H. (2018). Potential Use of Amniotic Membrane—Derived Scaffold for Cerebrospinal Fluid Applications. Int. J. Mol. Cell Med..

[79] Deus I., Santos S., Custódio C., Mano J.F. (2021). Designing highly customizable human based platforms for cell culture using proteins from the amniotic membrane. Mater. Sci. Eng. C.

[80] Nouri M., Ebrahimi M., Bagheri T., Fatemi M.J., Najafbeygi A., Araghi S., Molaee M. (2018). Healing Effects of Dried and Acellular Human Amniotic Membrane and Mepitelas for Coverage of Skin Graft Donor Areas; A Randomized Clinical Trial. Bull. Emerg. Trauma.

[81] Wu Z., Liu X., Yuan D., Zhao J. (2018). Human acellular amniotic membrane is adopted to treat venous ulcers. Exp. Ther. Med..

[82] Xue S.L., Liu K., Parolini O., Wang Y., Deng L., Huang Y.C. (2018). Human acellular amniotic membrane implantation for lower third nasal reconstruction: A promising therapy to promote wound healing. Burn. Trauma.

[83] Kakabadze A., Mardaleishvili K., Loladze G., Karalashvili L., Chutkerashvili G., Chakhunashvili D., Kakabadze Z. (2017). Reconstruction of mandibular defects with autogenous bone and decellularized bovine bone grafts with freeze-dried bone marrow stem cell paracrine factors. Oncol. Lett..

[84] Hashemi S.S., Mohammadi A.A., Moshirabadi K., Zardosht M. (2021). Effect of dermal fibroblasts and mesenchymal stem cells seeded on an amniotic membrane scaffold in skin regeneration: A case series. J. Cosmet. Dermatol..

[85] Hashemi S.S., Mohammadi A.A., Kabiri H., Hashempoor M.R., Mahmoodi M., Amini M., Mehrabani D. (2019). The healing effect of Wharton’s jelly stem cells seeded on biological scaffold in chronic skin ulcers: A randomized clinical trial. J. Cosmet. Dermatol..

[86] Greaves N.S., Iqbal S.A., Hodgkinson T., Morris J., Benatar B., Alonso-Rasgado T., Baguneid M., Bayat A. (2015). Skin substitute-assisted repair shows reduced dermal fibrosis in acute human wounds validated simultaneously by histology and optical coherence tomography. Wound Repair Regen..

[87] Guler S., Aslan B., Hosseinian P., Aydin H.M. (2017). Supercritical Carbon Dioxide-Assisted Decellularization of Aorta and Cornea. Tissue Eng. Part C Methods.

[88] Amensag S., McFetridge P.S. (2014). Tuning scaffold mechanics by laminating native extracellular matrix membranes and effects on early cellular remodeling. J. Biomed. Mater. Res. Part A.

[89] Bankoti K., Rameshbabu A., Datta S., Roy M., Goswami P., Roy S., Das A., Ghosh S., Dhara S. (2020). Carbon nanodot decorated acellular dermal matrix hydrogel augments chronic wound closure. J. Mater. Chem. B.

